# Uncovering novel loci and developing functional Kompetitive Allele Specific PCR markers for chilling requirement in peach via genome-wide association study

**DOI:** 10.1093/hr/uhag069

**Published:** 2026-03-05

**Authors:** Juan Yan, Jianlan Xu, Jiyao Li, Zhixiang Cai, Zheng Chen, Binbin Zhang, Shaolei Guo, Yuanyuan Zhang, Lei Guo, Ruijuan Ma, Mingliang Yu, Zhijun Shen

**Affiliations:** Institute of Pomology, Jiangsu Academy of Agricultural Sciences/Jiangsu Key Laboratory for Horticultural Crop Genetic Improvement, No. 50 Zhongling Street, Nanjing 210014, China; Institute of Pomology, Jiangsu Academy of Agricultural Sciences/Jiangsu Key Laboratory for Horticultural Crop Genetic Improvement, No. 50 Zhongling Street, Nanjing 210014, China; Institute of Pomology, Jiangsu Academy of Agricultural Sciences/Jiangsu Key Laboratory for Horticultural Crop Genetic Improvement, No. 50 Zhongling Street, Nanjing 210014, China; College of Horticulture, Nanjing Agricultural University, No. 1 Weigang, Nanjing 210095, China; Institute of Pomology, Jiangsu Academy of Agricultural Sciences/Jiangsu Key Laboratory for Horticultural Crop Genetic Improvement, No. 50 Zhongling Street, Nanjing 210014, China; Institute of Pomology, Jiangsu Academy of Agricultural Sciences/Jiangsu Key Laboratory for Horticultural Crop Genetic Improvement, No. 50 Zhongling Street, Nanjing 210014, China; Institute of Pomology, Jiangsu Academy of Agricultural Sciences/Jiangsu Key Laboratory for Horticultural Crop Genetic Improvement, No. 50 Zhongling Street, Nanjing 210014, China; Institute of Pomology, Jiangsu Academy of Agricultural Sciences/Jiangsu Key Laboratory for Horticultural Crop Genetic Improvement, No. 50 Zhongling Street, Nanjing 210014, China; Institute of Pomology, Jiangsu Academy of Agricultural Sciences/Jiangsu Key Laboratory for Horticultural Crop Genetic Improvement, No. 50 Zhongling Street, Nanjing 210014, China; Institute of Pomology, Jiangsu Academy of Agricultural Sciences/Jiangsu Key Laboratory for Horticultural Crop Genetic Improvement, No. 50 Zhongling Street, Nanjing 210014, China; Institute of Pomology, Jiangsu Academy of Agricultural Sciences/Jiangsu Key Laboratory for Horticultural Crop Genetic Improvement, No. 50 Zhongling Street, Nanjing 210014, China; Institute of Pomology, Jiangsu Academy of Agricultural Sciences/Jiangsu Key Laboratory for Horticultural Crop Genetic Improvement, No. 50 Zhongling Street, Nanjing 210014, China; Institute of Pomology, Jiangsu Academy of Agricultural Sciences/Jiangsu Key Laboratory for Horticultural Crop Genetic Improvement, No. 50 Zhongling Street, Nanjing 210014, China

## Abstract

Chilling requirement (CR) is a key determinant for bud dormancy release in peach [*Prunus persica* (L.) Batsch]. To examine the genetic basis of CR and facilitate the breeding of climate-resilient varieties, we conducted a genome-wide association study (GWAS) on a diverse panel of 213 peach accessions with their CR phenotypes. The CR phenotypic data collected over 3 years demonstrated high heritability (*H*^*2*^ = 0.86), indicating a strong genetic component. The GWAS analysis identified 52 SNPs associated with CR traits, with major loci clustered on chromosome 1 (17.3–21.2 and 43.7–47.3 Mb) and chromosome 2 (5.2–13.9 Mb), thereby both confirming established loci in the DAM cluster and identifying novel genetic regions. By focusing on regions exhibiting stable CR associations across years and which could be successfully validated by Kompetitive Allele Specific PCR (KASP) assays, 13 candidate CR-related genes were identified. Two highly robust KASP markers derived from loci in chromosome 1 were developed and validated. These markers effectively discriminated between low (<400 h) and high (≥900 h) CR phenotypes. The combined use of these two markers achieved 95.5% accuracy in identifying extreme low-CR phenotypes (CR < 300 h) in peach accessions. The identification of genes linked to these robust markers of CR-related loci and the analysis of their expression during dormancy identified three potentially related with CR trait modulation: a receptor-like protein kinase, a protein kinase and a BED-type zinc finger domain-containing protein. This study provides useful molecular tools for marker-assisted breeding for low-CR peaches and new insights into the complex regulatory network of CR.

## Introduction

Peach [*Prunus persica* (L.) Batsch] is one of the most economically important deciduous fruit trees globally. Its growth, flowering, and fruit yield are heavily dependent on chilling requirement (CR), the period of exposure to chilling conditions necessary for the release from bud dormancy and the initiation of spring growth. Adequate CR is critical for synchronized budburst, timely flowering, and proper fruit development, while insufficient chilling leads to delayed flowering, uneven budburst, and poor fruit quality, which ultimately reduces yield [1, 2]. CR varies significantly among cultivars, ranging from 0 to over 1500 h, reflecting its complex genetic regulation [3, 4].

Global climate change has led to increasingly milder winters, which leads to sub-optimal productivity from the cultivars currently used in the peach industry [4–6]. To address this challenge and facilitate the breeding of climate-resilient varieties, it is imperative to gain a deeper understanding of the genetic basis of CR and develop precise molecular markers for marker-assisted selection of low-CR genotypes. Consequently, these objectives have become top priorities in peach breeding programs. CR is a complex quantitative trait controlled by multiple genes and significantly influenced by environmental factors. The regulatory pathways in dormancy and chilling accumulation involve a complex interplay of photoperiod, low temperature signals, and hormonal changes, with central roles played by MADS-box family genes, especially the Dormancy-Associated MADS-box (DAM) genes [7–9], among which *PpDAM5* and *PpDAM6* are the most well characterized [3, 10–12]. The regulation of CR by *PpDAM6* occurs with its reduced expression during bud dormancy release and its interaction at the protein level with the mitogen-activated protein kinase, *PpMAPK6*, which shows opposite expression patterns in peach cultivars with varying CR. This interaction is influenced by abscisic acid (ABA) and is considered crucial for regulating bud dormancy release in peach [9].

Beyond the well-established transcriptional framework governed by *PpDAM5* and *PpDAM6*, it is increasingly recognized that the precise determination of CR involves a sophisticated, multi-layered regulatory network. At the forefront of this complexity is epigenetic regulation. Seminal studies have elucidated how prolonged cold is transduced into a stable ‘epigenetic memory’ via pathways such as CK2 kinase-PRC2, leading to genome-wide repressive marks, such as *H3K27* trimethylation [13]. Directly exemplifying this in peach, chilling temperatures dynamically induce such epigenetic modifications at specific DAM loci, orchestrating their downregulation [14]. Critically, these histone modifications at dormancy-associated genes directly influence the metabolism and signaling of phytohormones, such as ABA and gibberellin, thereby integrating environmental cues into the dormancy cycle [15]. This mechanistic link underscores that systemic hormonal changes constitute another critical layer, as evidenced by our recent comparative metabolomics showing distinct global phytohormone profiles between high- and low-CR varieties [16]. Furthermore, at the post-transcriptional level, factors such as specific microRNAs (e.g. miR6390 in pear) directly target and degrade DAM transcripts [17]. Most recently, plasmodesmata gating and specific hormone receptor complex formation have been indicated as essential for dormancy cycling [18, 19]. This intricate, multi-tiered regulatory network underscores the profound complexity of CR.

However, this very complexity poses a major practical challenge to the accurate phenotyping of CR, which is confounded by significant genotype–environment interactions.

The identification of specific QTLs and candidate genes associated with CR has been greatly accelerated by high-throughput sequencing technologies and genome-wide association studies (GWAS) [12, 20]. Significant QTLs linked to CR have been mapped to chromosomes 1, 4, 6, and 7 of the peach genome, with the most prominent region located between 34.8 and 47.4 Mbps on Chr01, which contains the *PpDAM5* and *PpDAM6* genes [3, 10–12]. However, to date, relatively few studies have investigated the role of non-MADS-box genes in CR regulation and our understanding of the genetic architecture underlying this trait remains incomplete.

The identification and detailed analysis of CR-related loci and genes have led to the development of several molecular markers to assist in marker-assisted selection of CR genotypes, including high-resolution melting [21] and Kompetitive Allele Specific PCR (KASP) markers [22]. Additionally, Zhao *et al*. [9] identified a 30-bp deletion in the promoter region of *PpDAM6* and established a PCR-based marker that can distinguish high-CR (>500 h) from low-CR (<500 h) cultivars. However, in our preliminary experiments, these previously reported KASP markers showed poor performance in populations of peach from southern China, underscoring the genetic complexity of CR and the need for discovery using a larger, more genetically diverse germplasm collection.

This study aims to (i) identify novel CR-associated SNPs and candidate genes across a diverse selection of peach cultivars using GWAS and expression analyses, and (ii) develop and validate robust, widely applicable KASP markers for marker-assisted selection of CR traits. By bridging genetic insights with practical applications, this research seeks to provide useful tools for breeding climate-resilient peach cultivars and to deliver new genetic insights into the complex regulatory network that define CR traits during bud dormancy.

## Results

### CR phenotypes are highly reproducible across years and show strong heritability

Our analysis of CR phenotypes in a genetically diverse panel of peach indicated that CR is a highly heritable and stable trait across years. Phenotypic evaluations of CR were conducted with 306 accessions consisting of 294 *P. persica* varieties and closely related *Prunus* species (hereafter referred to as peach accessions for simplicity). Of these, the average CRs of 287 accessions were determined across 3 to 5 years and could be categorized into accessions with low CR (CR < 400 h; *n* = 39, in which 22 peaches had a CR < 300 h), and mid-high CR (CR ≥ 400 h; *n* = 248, in which 22 peaches had a CR ≥ 900 h).

**Figure 1:**
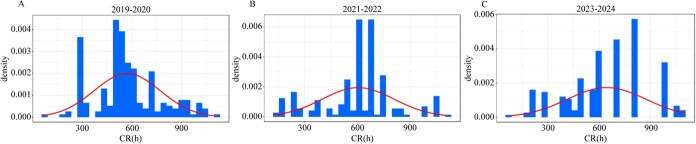
Histogram of CR phenotypes across three seasons of testing. (A) 2019–2020, (B) 2021–2022, (C) 2023–2024. The peach varieties analyzed were those used for GWAS.

The summary statistics of the CR phenotype data are presented in Table 1, which indicates substantial variation both between accessions and across years. Although Kolmogorov–Smirnov tests showed that data from most years deviated from a normal distribution (*P* < 0.05), the datasets used for GWAS were sufficiently symmetrical (Fig. 1A–C), with skew and kurtosis values <1, which can be considered acceptable for parametric analysis. To evaluate phenotypic reproducibility, Paired Pearson correlations of CR values from 213 core accessions between the 3 years of testing was performed, which indicated positive (*r* = 0.87–0.93) and highly significant (*P* < 0.01) correlations between each year. These high correlation coefficients indicate that the relative ranking of accessions based on CR were largely reproducible. Furthermore, the estimation of broad-sense heritability revealed a high genetic component (*H^2^* = 0.86), confirming that a substantial portion of the phenotypic variation is attributable to genetic differences (Table 1). In summary, the high heritability and strong phenotypic correlation across years provide a reliable basis for associating significant SNP loci with CRs through GWAS analysis.

**Table 1:** Summary statistics of CR phenotypic data.

Group	Group size	Year	CR range (h)	Mean ± SD (h)	Skewness	Kurtosis	Kolmogorov–Smirnov Test, *P*
Phenotypic Evaluation Group	83	2018–2019	234–1264	671 ± 284	0.28	−1.19	<0.05
	306	2019–2020	65–1108	599 ± 194	−0.1	−0.31	<0.05
	215	2021–2022	119–1104	607 ± 205	−0.25	0.31	<0.05
	175	2022–2023	153–1200	675 ± 221	0.43	0.19	>0.05
	300	2023–2024	82–1100	667 ± 214	−0.32	−0.39	<0.05
GWAS Analysis Group	213	2019–2020	65–1108	595 ± 205	0.07	−0.33	<0.05
		2021–2022	119–1104	626 ± 223	0.07	−0.55	>0.05
		2023–2024	82–1100	637 ± 234	−0.51	−0.72	<0.05
Validation Large Group	287	2018–2024	126–1112	640 ± 186	−0.41	0.34	<0.05

### GWAS of peach SNPs with CR phenotypes

**Figure 2:**
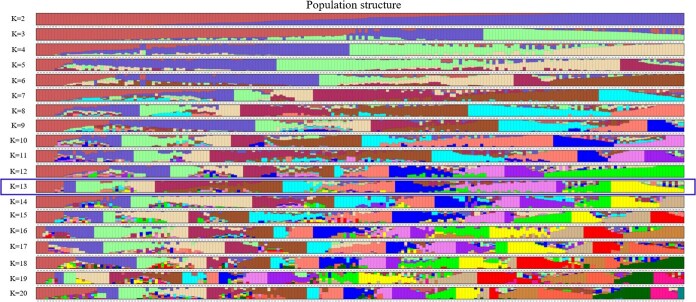
Population structure analysis in the GWAS population. Each row represents a distinct value of *K* (ranging from 2 to 20), corresponding to the *modeled* number of ancestral populations. Individuals are arranged along the horizontal direction, with each vertical column representing one sample. Colored segments within a column show the estimated ancestry proportion for each individual. The optimal model (*K* = 13), was determined by minimizing the cross-validation error, and is outlined in blue.

Prior to GWAS, population genetic analyses were conducted to ensure data quality. Population structure analysis revealed an optimal genetic clustering at *K* = 13 (Fig. 2, see also cross-validation in Fig. S1A), indicating substantial diversity. Linkage disequilibrium (LD) decayed rapidly, with the average *r*^2^ value falling below 0.1 within 100 kb (Fig. S1B), confirming adequate marker density for association mapping. Kinship analysis further confirmed that the panel is primarily composed of distantly related accessions (Fig. S1C). These results established a robust foundation for association analysis. After filtering for quality, a total of 1 304 248 high-quality SNP markers were retained for GWAS analysis.

**Figure 3:**
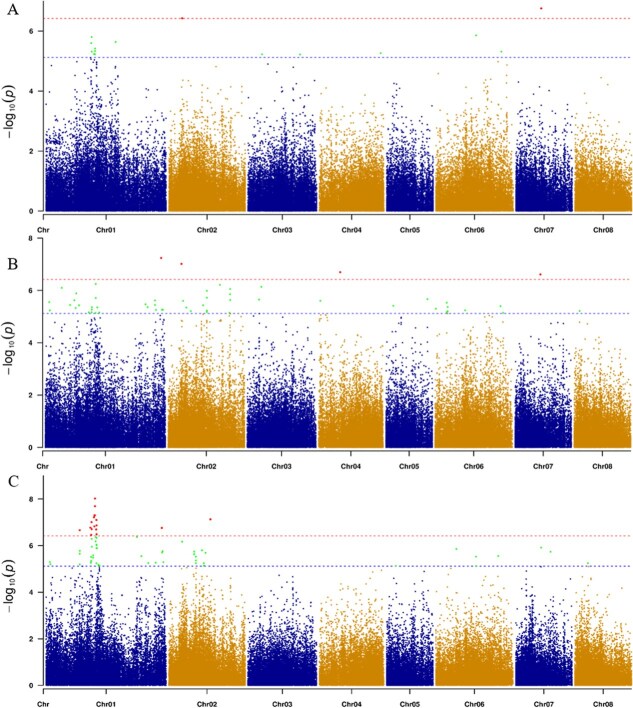
Manhattan plots of CR GWAS analysis in different years. (A–C) 2019–2020, 2021–2022, and 2023–2024, respectively. Each panel displays subplots for chromosomes 1–8, with the *x*-axis depicting genomic positions (in Mb). The y-axis represents the association strength of SNPs with CR phenotype as −log₁₀(*P* value). The red dashed line represents the threshold for SNP significance after Bonferroni correction (*P* = 0.05/total SNPs, equivalent to −log_10_(*P*) = 6.42, corresponding to *P* = 3.80 × 10^−7^). The blue dashed line represents the threshold for associations (*P* = 1/total SNPs, equivalent to −log_10_(*P*) = 5.12, corresponding to *P* = 7.59 × 10^−6^). SNPs with *P* values above these thresholds are colored red and green, respectively.

The association of SNPs with CR phenotypic data was tested with general linear model (GLM) and mixed linear model (MLM). The MLM proved to be too conservative (data not shown), failing to identify several previously reported associations [3, 10]. Therefore, we present the results from GLM (Fig. 3A–C). The SNP density across chromosomes 1 to 8 and quantile–quantile (Q–Q) plots are shown in Figs S2 and S3, respectively. A slight overall upward shift across all seasons indicates mild genomic inflation, which is consistent with the detectable population stratification (K = 13). In total, 117 SNPs reached the suggestive threshold (*P* < 7.59 × 10^−6^) for associations with CR traits across seven chromosomes over the three analyzed seasons. Among these, 22 SNPs surpassed the Bonferroni corrected (α = 0.05) significance threshold (*P* < 3.80 × 10^−7^) and were considered the most robust associations. Of the total 117 CR-associated SNPs, 52 were selected for further study due to their location in gene coding or known regulatory regions (Table 2). Chr01 harbored 40 CR-associated SNPs, of which 10 were associated in at least two of the 3 years of testing. Chr02 harbored 15 unique SNPs, 2 of which showed CR-associated in at least 2 years. CR-associated SNPs were primarily located in the bottom (43.7–47.3 Mbps) and upper-middle (17.3–21.2 Mbps) regions of Chr01, but were also detected in the middle region of Chr02 (05.2–13.9 Mbps). The remaining chromosomes showed fewer CR-associated SNPs, with Chr08 displaying no associations (Table 2). Overall, 14 CR-associated SNPs that were detected in two or more seasons were selected as candidate loci for further validation (listed in Table 2).

**Table 2:** The 52 significant CR-related SNPs identified from the GWAS analysis.

Chr	Significant SNPs			Total SNPs (including duplicates)	SNPs detected at least two years (Number)	SNP location
	2019–2020	2021–2022	2023–2024			
Chr01	5	15	20	40	10	Chr01:1494349^*b, *c^; Chr01:1745668^*b, *c^; Chr01:1753441^*b, *c^; Chr01:13406158^*b, **c^; Chr01:17976375^*a, **c^; Chr01:19070198^*a, **c^; Chr01:19436628^*a, **c^; Chr01:19494698^*a, **c^; Chr01_20,053866^*b, *c^; Chr01_46,470 090^*b, *c^
Chr02	1	10	4	15	2	Chr02:5261859^**a, **b, *c^; Chr02:13915162^*b, *c^
Chr03	2	1	0	3	1	Chr03: 5579842^*a, *b^
Chr04	0	2	0	2	0	-
Chr05	0	1	0	1	0	-
Chr06	0	2	1	3	0	-
Chr07	1	1	1	3	1	Chr07:10063980^**a, **b, *c^
Chr08	0	0	0	0	0	-
Total	9	32	26	67	14	-

^a, b, c^ indicate which season/s of testing SNPs were detected as significant (2019–2020, 2021–2022, and/or 2023–2024, respectively). Asterisks denote the adjusted p-value thresholds utilized for significance testing: ^*^P < 7.59 × 10^−6^, ^**^P < 3.80 × 10^−7^.

### Evaluation of KASP marker utility for the prediction of CR phenotypes

A total of 20 KASP markers were designed including those for the 14 significant CR-associated SNP loci indicated above (Table 2). The other six were developed for SNPs that reached significance in only 1 year but are located within the previously reported CR-associated region on Chr01 (34.8–47.4 Mb) [22]. These included loci at Chr01:43694461, Chr01:43704672, Chr01:43706671, Chr01:44074507, Chr01:46282355, and Chr01:46766605. We included this latter group to evaluate whether SNPs in this key genomic region could still serve as useful markers in our diverse panel, despite their lower temporal reproducibility.

A preliminary evaluation of the KASP SNP markers was conducted using 24 peach cultivars with extreme CRs (high or low CR; Table S1). The analysis revealed that 10 of the 20 KASP markers showed significant associations with CR phenotypes (*P* < 0.05), with correlation coefficients ranging from 0.20 to 0.88. These markers exhibited high stability in both significance and correlation coefficients across the 3 years studied (Table 3). Most notably, the three markers from Chromosome 1 (Chr01:43704672, Chr01:43706671, and Chr01:46470090) showed consistently high statistical significance (*P* < 10^−6^) and robust associations with CR (correlation coefficients, 0.71–0.86). These findings highlight their stable predictive power across varying environmental conditions and indicates they are promising candidates for further investigation and application.

**Table 3:** Evaluation of candidate SNPs via KASP molecular markers

	Validation in 24 extreme samples						Validation in 287 samples	
	2019–2020		2021–2022		2023–2024		2018–2024	
SNP/KASP candidate marker	Sig. value	*R* ^2^	Sig. value	*R* ^2^	Sig. value	*R* ^2^	Sig. value	*R* ^2^
Chr01:1745668	0.01	0.34	0.026	0.29	0.01	0.32	4.65 × 10 ^−6^	0.08
Chr01:1753441	0.02	0.29	0.05	0.25	0.04	0.25	1.53 × 10 ^−5^	0.07
Chr01:13406158	0.04	0.27	0.028	0.3	0.03	0.28	0.72	4.40 × 10 ^−6^
Chr01:43694461	1.78 × 10 ^−4^	0.57	1.48 × 10 ^−4^	0.57	1.50 × 10 ^−4^	0.57	1.08 × 10 ^−13^	0.19
Chr01:43704672	4.92 × 10 ^−8^	0.88	6.13 × 10 ^−6^	0.85	7.9 × 10 ^−7^	0.84	1.86 × 10 ^−13^	0.18
Chr01:43706671	2.56 e × 10 ^−6^	0.82	3.67 × 10 ^−7^	0.86	8.04 × 10 ^−7^	0.84	7.04 × 10 ^−21^	0.28
Chr01:46282355	0.01	0.44	0.005	0.41	0.01	0.44	8.84 × 10 ^−10^	0.13
Chr01:46470090	4.03 × 10 ^−6^	0.7	1.98 × 10 ^−6^	0.71	2.53× 10 ^−6^	0.71	1.85 × 10 ^−21^	0.28
Chr01:46766605	0.03	0.3	0.017	0.33	0.02	0.32	2.3 × 10 ^−5^	0.07
Chr02:5261859	0.03	0.21	0.014	0.25	0.034	0.2	3.29 × 10 ^−9^	0.12

To further validate the reliability and efficacy of these 10 markers, they were tested against a broader genetic background of 287 accessions consisting of commercial cultivars, landraces and closely related wild species originating from various countries (Table S1). The results indicated that 9 out of the 10 markers showed significant associations with CR phenotypes (*P* < 10^−5^). The contribution of marker genotypes to CR variation, measured as the proportion (%) of explained variation in phenotype (R^2^), ranged from 7.4 to 28.6. Of these, the highest proportion of phenotypic variation could be explained by the presence of the SNPs Chr01:46470090 (28.6%) and Chr01:43706671 (28.0%). The primer sequences for these two KASP Markers are shown in Fig. S4. Importantly, their performance against this large population was consistent with that observed against cultivars with more extreme CRs, indicating their high potential for application in breeding programs.

To evaluate the discriminative capacity of these two KASP markers against peach genotypes of differing CR, accessions were stratified into CR groups ranging from extreme low (CR ≤ 300 h, *n* = 22), low (300 h < CR < 400 h, *n* = 39), mid-high (CR ≥ 400 h, *n* = 248) to extreme high (CR ≥ 900 h, *n* = 22).

**Figure 4:**
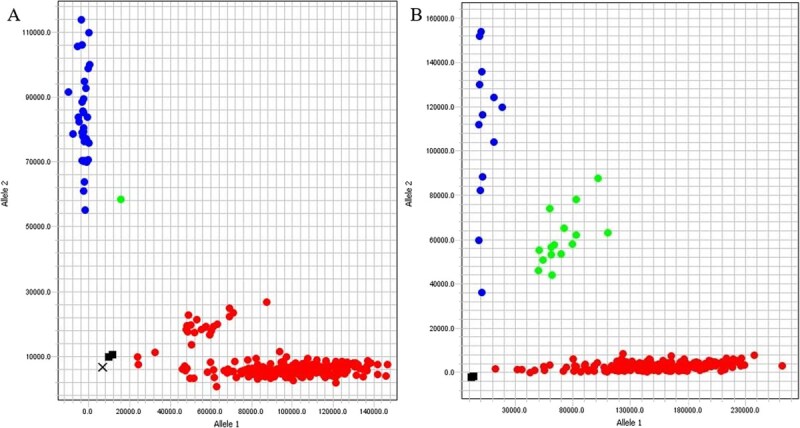
The use of KASP markers to assay the allelic diversity in peach accessions at SNP loci (A) Chr01: 43706671 and (B) Chr01:46470090. Each data point in the main panel represents a sample accession. The blue and red points represent SNP homozygotes for allele 1 and allele 2, respectively. The green points and black crosses represent SNP heterozygotes and undetermined zygosity, respectively. Black squares indicate negative control data. The positive controls confirmed by sanger sequencing were ‘Flordaglo’ with a GG genotype and ‘FC’ with a TT genotype (reverse sequencing) for Chr01: 43706671; ‘Flordaglo’ with a CC genotype and ‘FC’ with a TT genotype for (Chr01:46470090) were included in the above 287 materials and are not distinguished in the figure.

**Figure 5:**
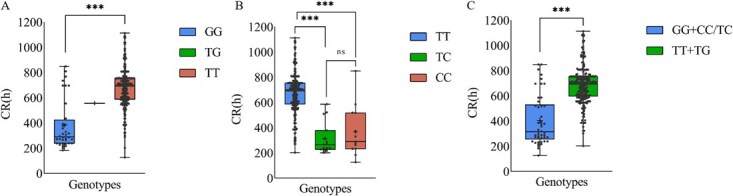
The distribution of CR (hours) with SNP allelic diversity in peach accessions at SNP loci (A) Chr01: 43706671 and (B) Chr01:46470090 and (C) A and B combined. Data for each genotypic group is presented as mean CR with error bars representing the ±SD. Individual dots represent the CR value of each accession. ^***^P < 0.001; ns, not significant.

The KASP marker for Chr01:43706671 distinguished three genotypes (TT, TG, GG) (Table S4), with one undetermined sample (Fig. 4A) subsequently confirmed as TT by Sanger sequencing. Mann–Whitney *U* tests indicated significant differences (*P* < 0.001) in the average CR between the GG (381 ± 191 h; *n* = 36) and TT genotypes (678 ± 153 h; *n* = 250) (Fig. 5A). Notably, all extreme high-CR accessions (CR ≥ 900 h, *n* = 22) exhibited TT homozygosity, confirming its dominance in high-CR phenotypes. A single TG heterozygote was identified with an intermediate CR value (556 h), which did not clearly fall within the range of either homozygous group. This suggests the possibility of incomplete dominance or the influence of other genetic backgrounds, though a larger sample size is needed to confirm this observation.

The KASP marker for Chr01:46470090 resolved TT, TC, and CC genotypes (Fig. 4B, Table S4). Both CC (*n* = 13; 367 ± 200 h) and TC (*n* = 15; 314 ± 126 h) showed significantly lower CR than TT homozygotes (*n* = 259; 673 ± 158 h, *P* < 0.001), with all high-CR accessions (≥900 h) being TT homozygous (Fig. 5B).

The combined use of these KASP markers resolved the accessions into low and mid-high CR groups (Fig. 5C; *P* < 0.001). The TG and TT genotypes at Chr01:43706671 combined with the TT genotype at Chr01:46470090 (n = 241; 685 ± 146 h) were associated with high CR, while those with GG (Chr01:43706671) and CC/TC (Chr01:46470090) (*n* = 46; 404 ± 200 h) were associated with low CR. Importantly, 95.5% of the extreme low-CR accessions (CR < 300 h, *n* = 22) were accurately classified into the GG and CC/TC cohort.

The predictive performance metrics of these markers are shown in Table 4, which indicates their complementary characteristics. Both maintained high specificity against mid-high CR phenotypes (≥400 h), with Chr01:43706671 at 96.4% and Chr01:46470090 at 97.2%, while their combination retained 93.5% specificity. Chr01:43706671 showed superior sensitivity (69.2%) compared to Chr01:46470090 (53.8%), while a balanced sensitivity (65.2%) was obtained with their combined use. The positive predictive value (PPV) increased from 75.0% with the use of individual markers to 76.9% when used in combination. The negative predictive values (NPVs) exceeded 93% with individual markers, but was also increased (96.3%) when used in combination. These findings establish Chr01:43706671 as a primary screening marker for low-CR genotypes. The combined use of both markers however improves detection coverage by incorporating 95.5% of extreme low-CR accessions (CR < 300 h) into the GG/CC/TC group without compromising high exclusion efficacy (NPV = 96.3%), thereby significantly enhancing the identification of low-CR germplasm.

**Table 4:** Performance of KASP markers in predicting low CR phenotypes.

Metric	Chr 01:43706671	Chr 01:46470090	Combined markers
Sensitivity (%)^a^	69.2 (GG)	53.8 (CC/TC)	65.2 (GG/CC/TC)
Specificity (%)^b^	96.4 (TT/TG)	97.2 (TT)	93.5 (TT)
PPV^c^ (%)	75.0	75.0	76.9
NPV^d^ (%)	95.2	93.1	96.3

a Sensitivity, proportion of true low-CR samples correctly identified.

b Specificity, proportion of true mid-high CR samples correctly excluded.

c PPV, probability that predicted low-CR samples are truly low-CR.

d NPV, probability that predicted mid-high CR samples are truly mid-high CR.

### KASP marker-associated gene analysis and candidate gene identification

Considering the decay distance indicated in the LD analysis, regions of 150 kb flanking the 9 CR phenotype-associated SNPs (indicated in Table 3) were selected to identify potentially associated genes. A total of 13 genes were identified as potentially linked to these significant SNPs (Table 5).

**Table 5:** The candidate CR-related genes associated with significant SNPs. Note that the loci Chr01:43704672 and Chr01:43706671 are associated with the same gene, Prupe.1G534800 (Gene number 4), and are located in exons and downstream regions from the gene, respectively.

SNPs number	SNP/KASP candidate marker	Genes number	Associated gene	Genomic region of SNP in associated gene
1	Chr01:1745668	1	Prupe.1G025200	Intronic
2	Chr01:1753441	2	Prupe.1G025400	Upstream
3	Chr01:43694461	3	Prupe.1G534600	Downstream
4	Chr01:43704672	4	Prupe.1G534800	Exonic
5	Chr01:43706671	4	Prupe.1G534800	Downstream
		5	Prupe.1G534900	Upstream
6	Chr01:46282355	6	Prupe.1G567000	Upstream
		7	Prupe.1G567100	Downstream
7	Chr01:46470090	8	Prupe.1G570300	Upstream
		9	Prupe.1G570400	Upstream
8	Chr01:46766605	10	Prupe.1G573900	UTR3
		11	Prupe.1G574000	UTR3
9	Chr02:5261859	12	Prupe.2G046100	Downstream
		13	Prupe.2G046200	Downstream

### Identification of differentially expressed CR-associated genes

To validate the candidate genes, their expression dynamics during endodormancy were compared by qRT-PCR in high- and low-CR cultivars. A stepwise statistical filtering strategy based on large-effect thresholds (see Materials and methods) was then applied to identify core candidates with robust associations to CR variation. This evaluation is summarized in Table 6. Of the 13 candidates evaluated, only three genes, Prupe.1G025200, Prupe.1G570300, and Prupe.1G534800, satisfied all criteria for significance, effect size and within-group consistency, and were thus designated as the core candidate genes (Table 6). Each exhibited a significant differential response between CR groups across dormancy stages, characterized by a strong ‘group × stage’ interaction, a large between-group difference in expression and highly synchronized expression trends within each CR group. Notably, Prupe.1G534800 and Prupe.1G570300 are linked to the two most robust SNP markers identified above (Chr01:43706671 and Chr01:46470090), which also accounted for the highest proportion of phenotypic variance in CR (*R*^2^ > 0.28).

**Table 6:** Statistical evaluation of the CR association of candidate genes in peach.

Gene ID	LMM: Group × Stage interaction *P*-value/Partial η^2^	Stage transition effect size (|Cohen’s *d*|) Stage 2 vs 1/Stage 3 vs 2	Intra-group consistency (Pearson *r*) Low-CR/High-CR	Core candidate genes
Prupe.1G025200	0.013/0.581	0.64/14.14	0.83/0.99	Yes
Prupe.1G025400	0.842/0.018	0.74/0.82	−0.41/0.36	No
Prupe.1G534600	0.567/0.045	0.44/0.48	0.79/0.62	No
Prupe.1G534800	0.003/0.217	2.26/1.4	0.84/0.77	Yes
Prupe.1G534900	0.014/0.075	0.20/1.70	0.99/0.95	No
Prupe.1G567000	0.234/0.103	0.65/0.65	0.51/−0.36	No
Prupe.1G567100	0.198/0.115	0.65/1.30	0.99/−0.25	No
Prupe.1G570300	0.037/0.388	0.24/4.96	0.97/−0.94	Yes
Prupe.1G570400	0.312/0.082	0.98/0.65	0.74/−0.19	No
Prupe.1G573900	0.423/0.062	0.32/3.63	0.69/0.73	No
Prupe.1G574000	0.278/0.091	1.81/1.74	0.96/0.59	No
Prupe.2G046100	0.067/0.187	0.98/1.30	0.43/0.96	No
Prupe.2G046200	0.134/0.142	0.98/3.41	−0.06/0.89	No

The remaining 10 candidate genes (Fig. S5) did not fulfill all criteria, primarily due to insufficient within-group consistency (reflecting considerable inter-cultivar variability), weak between-group effect sizes, or both (Table 6). These were not considered further in this study, though their potential functional roles in CR regulation were not excluded.

**Figure 6:**
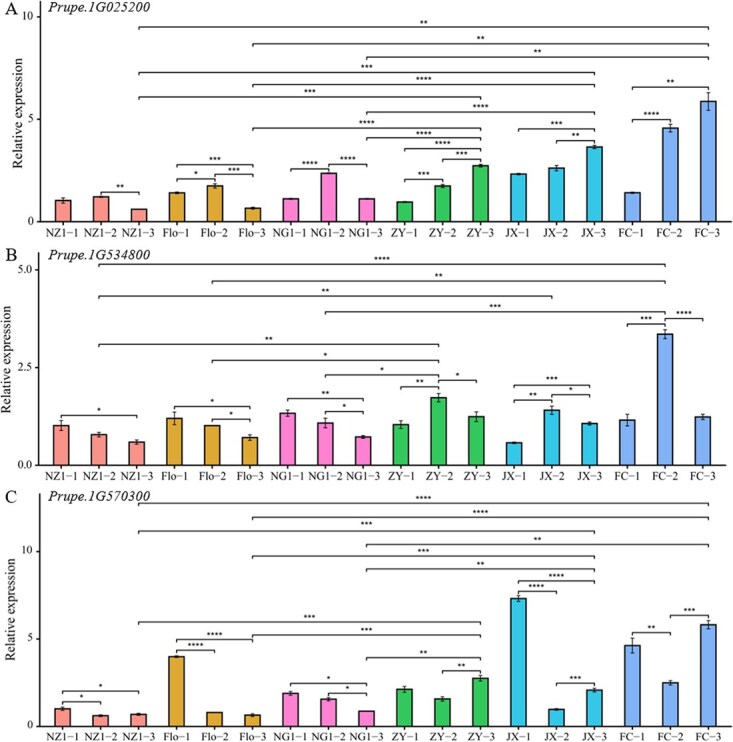
The relative expression levels of three CR-associated genes in peach varieties of different CRs. (A) *Prupe.1G025200*; (B) *Prupe.1G534800*; (C) *Prupe.1G570300*. The *x*-axis labels describe the low CR cultivars, NZ1, Flo, NG1, and the high CR cultivars, ZY, JX, and FC. The additional numeral in suffix (1–3) represents the different stages of endodormancy: onset, mid-endodormancy and dormancy release, respectively. Genes expression is shown relative to that in the Low-CR cultivar, NZ1, at stage 1, the onset of endodormancy. Data are presented as mean ± SE. Statistically significant differences between data points are indicated with asterisks: ^*^P < 0.05; ^**^P < 0.01; ^***^P < 0.001; ^****^P < 0.0001.

The expression profiling of the three core candidates demonstrated their distinct dynamics during the progression of bud endodormancy in representative high- and low-CR varieties (Fig. 6). *Prupe.1G025200* demonstrated an increase at mid-endodormancy in low-CR lines, but increased progressively in high-CR lines, achieving significantly higher expression in high-CR varieties at dormancy release (stage 3; *P* < 0.01). Prupe.*1G534800* exhibited a general decline in low-CR lines but peaked at mid-dormancy (Stage 2) in high-CR lines. Overall, expression at this stage was significantly elevated in the high-CR group compared to the low-CR group (*P* < 0.05), although some variation was observed between cultivars. Prupe.1G570300 progressively decreased in low-CR lines, but was more complex in high-CR lines, resulting in significantly higher expression at dormancy release (stage 3; *P* < 0.01).

*Prupe.1G025200* and *Prupe.1G570300* both encode protein kinases with a canonical protein kinase domain (IPR000719) within the protein kinase-like domain superfamily (IPR011009), which is associated with ATP-binding and protein phosphotransferase activity. However, Prupe.1G570300 is also predicted to be a transmembrane receptor-like kinase with serine/threonine kinase activity, suggesting roles in signal transduction and responses to the environment. *Prupe.1G534800* is predicted to encode a BED-type zinc finger domain-containing protein, homologous to members of the RICESLEEPER family (e.g. *Theobroma cacao* RICESLEEPER 2-like, with which it shows 58% identity). Together, these results suggest that further study of these three genes may provide further insights into the regulatory network governing CR.

## Discussion

### Novel CR loci identified by GWAS against a germplasm collection enriched in low-CR cultivars from Southern China

The power of GWAS to indicate the associations of genes or loci with traits requires accurate phenotyping and that the genotypic basis for the phenotypes under study is sufficiently represented within the population under investigation. While several CR-associated markers have been reported, their validation has often been confined to narrow populations or genotypes selected from specific environmental conditions, which can limit their broader utility [21]. KASP markers developed by Demirel *et al*. [22] failed to distinguish low CR phenotypes in our more diverse panel of 287 accessions (data not shown). The recently developed *PpDAM6* promoter markers for CR [9] were selected from predominantly temperate cultivars, where only 3.8% and 1.4% of accessions had a CR of <600 h and < 400 h, respectively, which might reduce their effectiveness against low CR phenotypes.

To facilitate the identification of loci associated with low CR phenotypes, the large germplasm collection utilized in this study was enriched with low–CR-associated accessions from Southern China (a subtropical climate), so that the proportions of cultivars with mid and low CR were increased to 46.0% (CR < 600 h) and 17.8% (CR < 400 h), respectively. This enrichment ensured the necessary genetic diversity to uncover alleles that are likely to be low frequency or monomorphic in temperate germplasm panels.

Over five consecutive years, we conducted 1077 CR phenotype assays. To ensure the accuracy of these, the chilling accumulation model was optimized for the local climate in Nanjing, which is characterized by fluctuating winter temperatures that can undermine the stability of standard models. The 0°C to 7.2°C model was selected for its consistent performance under these conditions. The high reproducibility of the resulting data as evidenced by strong inter-annual correlations (r = 0.87–0.93) and a high *H^2^* (0.86) indicated that the CR phenotypic data could be used to test their associations with genetic loci with confidence.

The associations indicated in this study included loci within chromosome 1 (43.7–47.3 Mbps) that have been reported to be CR-related [3, 8, 10], which supports the validity of our data and approach. However, this study also identified novel clusters of significant SNPs in the upper-middle region of chromosome 1 (17.3–21.2 Mbps) and in chromosome 2 (5.2–13.9 Mbps), which are likely to be low-frequency or monomorphic in germplasm panels of temperate peach cultivars [9, 12, 13].

### Development and validation of molecular markers for CR in peach

We have developed and rigorously validated two KASP markers (Chr01:43706671 and Chr01:46470090) against a panel of 306 accessions. These markers exhibited highly significant associations with CR variation. The strong explanatory power of these markers was evidenced by the fact that all the extreme high-CR (≥900 h) accessions were TT homozygous at both loci. The marker Chr01:43706671 alone serves as an excellent first-pass filter for low-CR phenotypes, balancing high specificity (96.4%) with considerable sensitivity (69.2%). However, the combined use of these markers efficiently identified the relatively rarer accessions with low-CR traits. The initial selecting for the GG genotype at Chr01:43706671 and the subsequent selection for CC/TC genotypes at Chr01:46470090 captured 95.5% of the extreme low-CR (<300 h) germplasm.

The advantage from the combined use of these markers is the exceptionally high NPV (96.3%). This indicates the reliable exclusion of varieties with CR traits outside of the desirable low range with great confidence, a feature of particular value to breeders in low-latitude regions aiming to introgress low-CR alleles.

Nevertheless, the moderate overall sensitivity (65.2%) for the less extreme low-CR phenotypes (<400 h) suggests that additional genetic factors remain to be identified. Future work should focus on integrating markers from other regions, such as upper-middle region of chromosome 1 and chromosome 2, to achieve a more comprehensive coverage.

### Novel insights into the genetic regulation of peach CR

Our study reinforces the emerging consensus that CR is a classic polygenic trait. While Dormancy-Associated MADS-box (DAM) genes, particularly *PpDAM5* and *PpDAM6*, have been established as master regulators of endodormancy progression [7–9], the GWAS did not identify them as major effect loci. This can be expected and attributed to population genetic factors, such as limited haplotype diversity or the selection against deleterious alleles at these core loci in breeding materials, which can reduce their association with phenotypic variation [13]. Our data confirmed the expression of the DAM genes, *PpDAM4*, *PpDAM5*, and *PpDAM6* is closely associated with dormancy progression, which agrees with earlier studies [7, 8]. Nevertheless, the expression profiles of these DAMs were highly similar between high- and low-CR cultivars (Fig. S6A), suggesting that their transcriptional regulation alone cannot explain the phenotypic differences in CR, which must require additional modulatory genes or post-transcriptional mechanisms for their expression.

The integration of the GWAS data with temporal expression profiling indicated three strong candidate genes which exhibited differential expression patterns between low- and high-CR cultivars during the dormancy period (Fig. 5). Linked to the robust SNP marker Chr01:46470090, Prupe.1G570300 encodes a receptor-like kinase that was significantly upregulated in high-CR cultivars during endodormancy release. Further examination of the relationship this kinase shows with known signaling pathways operating during bud dormancy, such as ABA- and GA-related signaling [23, 24], may provide further insight into RLK roles in CR determination. Prupe.1G534800 is linked to the robust SNP marker Chr01:43706671 and encodes a BED-type zinc finger domain-containing protein. Phylogenetically, it belongs to the SLEEPER family of domesticated transposase-derived transcriptional regulators, which are essential for plant development [25, 26]. Investigation of the regulatory targets of *Prupe.1G534800* during dormancy may therefore provide further insight into the regulation of dormancy and/or CR traits. *Prupe.1G025200*, which is located in the novel CR Loci within the upper-middle region of chromosome 1 (17.3–21.2 Mbps), is a member of the serine/threonine protein kinase family. Its differential expression across dormancy stages suggests that it is functionally associated with dormancy progression, where its phosphotargets may contribute to the establishment of stage-specific dormancy characteristics and/or the regulation of the transitions between dormancy stages.

To investigate the association of these three candidate genes with the known DAM-mediated dormancy regulatory network, we analyzed their co-expression relationships with DAM genes (*PpDAM1–6*). The results indicated that only the BED-zinc finger gene, *Prupe.1G534800*, showed significant positive co-expression with *PpDAM3*, *PpDAM4*, *PpDAM5*, and *PpDAM6* (*P* < 0.05; Fig. S6B). This co-expression indicates that *Prupe.1G534800* is co-regulated with core elements in the dormancy regulatory network, suggesting it may function as a regulatory partner of DAMs, or as a modulator or effector within the DAM gene network. In contrast, the receptor kinase Prupe.*1G570300* and the serine/threonine protein kinase *Prupe.1G025200* showed no significant co-expression with the DAM genes, which is more consistent with their roles in parallel or convergent pathways for dormancy modulation, potentially fine-tuning the CR response independently of, or in coordination with, the DAM-mediated transcriptional program.

The evidence from genetic (GWAS), marker-based (KASP), and transcriptional studies together suggests that these three genes are involved in the regulation of dormancy and the definition of CR traits. We therefore propose that these genes be targeted for further studies to gain insight into the control of CR in peach.

## Conclusions

This study provides further insights into the genetic regulation of CR traits in peach by integrating GWAS, functional marker development, and candidate gene analyses. We confirmed the highly heritable nature of CR (*H^2^* = 86.39%) and identified 52 significant SNPs, with major novel loci on chromosomes 1 and 2, including novel loci unrelated to known DAM genes. From selected loci, we developed two highly robust KASP markers (Chr01:43706671 and Chr01:46470090) that enable accurate selection of extreme low-CR genotypes (<300 h) with 95.5% accuracy, providing a powerful tool for breeding climate-resilient cultivars.

The integrated analysis of GWAS, expression profiling (in representative high- and low-CR varieties), and functional marker validation identified three candidate genes for CR variation: a receptor-like protein kinase (*Prupe.1G570300*), a protein kinase (*Prupe.1G025200*), and a BED-type zinc finger domain-containing protein (*Prupe.1G534800*). Based on this multi-layered evidence, we propose that these genes, in addition to the core DAM pathway, are likely involved in modulating bud dormancy progression and thereby influence CR.

This work provides both actionable molecular tools for CR trait selection in peach breeding programs and novel mechanistic insights into CR trait regulation. Future efforts should now focus on the functional validation of these candidate genes and the exploration of their involvement in gene–environment interactions to further advance the development of peach varieties with CR adapted to climate change.

## Materials and methods

### Plant materials

All plant materials were obtained from the National Peach Fruit Germplasm Repository in Nanjing, China (32°20′N, 118°52′E, 11 m above sea level) from November 2018 to March 2024. The trees were grown under standard orchard management practices. A diverse panel of 306 accessions was used for the GWAS. The data were collected between November 2018 and March 2024. This collection comprised 294 varieties of *Prunus persica* and 12 accessions from closely related *Persica* species, including *Prunus ferganensis* (6), *Prunus davidiana* (4), *Prunus kansuensis* (1), and *Pyrus communis* Fritsch (1) (listed in Table S1). For simplicity, all accessions are hereafter referred to as peach.

Gene expression analysis were conducted during the 2024 to 2025 dormant season using six peach cultivars with contrasting CRs: three low-CR varieties (‘NGT1,’ CR = 189 h; ‘Flordaglo’ (‘Flo’), CR = 232 h; and ‘NZ1’, CR = 283 h) and three high-CR varieties (‘ZY’, CR = 669 h; ‘JX,’ CR = 686 h; and ‘FC’, CR = 1037 h). Floral buds were sampled at the onset of endodormancy onset [chill accumulation (CA) = 0 h], mid-dormancy (CA ≈ 50% of CR), and at the release from dormancy (CA ≈ CR) as described by Zhang *et al*. [27]. Each sample consisted of three biological replicates, with each replicate comprising at least 30 floral buds. Samples were immediately flash-frozen in liquid nitrogen after collection and stored at −80°C until RNA extraction.

### Analysis of CR phenotypic data

The cultivation of peach twigs and data collection were performed essentially as described by Yan *et al*. [28] and Zhang *et al*. [27]. Briefly, the CR (hours) of each genotype was determined through budbreak forcing assays conducted in the greenhouse during the 2018 to 2024 seasons. Hourly average temperatures were recorded throughout the experimental periods to calculate the CA using the 0°C to 7.2°C model (i.e. the total hours of exposure to temperatures between 0°C and 7.2°C).

Five healthy 1-year-old twigs (30–40 cm in length) of each genotype were randomly selected from vigorous trees at intervals of 5 days after an initial accumulation of approximately 50 chill hours. After collection, the basal ends of the twigs were trimmed, placed into a 5% sucrose solution and subject to forced budbreak trials in a greenhouse under a 16 h photoperiod at 25°C (forcing conditions; described in full in Yan *et al*.) [28]. Bud break progression was evaluated after 12 days. The developmental stage of the buds was classified numerically as either dormant (i), green tip visible (ii), red tip visible (iii), flower bud stage (iv) or open flower (v). The weighted average score was calculated using: $\sum \limits_{i-1}^5 iXi/\sum \limits_i^5 Xi$, where *i* represents the numerical value assigned to the bud developmental stage, and Xi represents the number of buds at each stage. CR was considered fulfilled when the weighted average bud score was ≥2.5. To investigate environmental influences other than the chilling conditions (0°C–7.2°C), the CR was determined annually from 2018 to 2024 for all peach accessions (Table S1).

The normality of the CR distribution for each year was assessed using the Kolmogorov–Smirnov test. To evaluate the reproducibility and year-to-year stability of CR traits, the CR values of the 213 accessions from each year of testing (2021–2022, 2022–2023, and 2023–2024) were subjected to paired Pearson correlation analysis. Broad-sense heritability (*H^2^*) of GWAS datasets was calculated on an entry-mean basis. The genetic (σ^2^g) and residual (σ^2^ε) variance components were derived from the mean squares of an ANOVA with the accession and year as random factors. The calculation followed the standard formula: *H^2^* = σ^2^g / (σ^2^g + σ^2^ε/*n*), where n is the number of testing years.

### The use of GWAS to identify SNPs linked with CR phenotypes

This study used whole-genome Illumina resequencing data of 213 peach accessions generated in our earlier research (unpublished data), with a sequencing depth of 30×. The genomic sequences were aligned to the *Prunus persica* Whole Genome Assembly v2.0 (obtained from the genome database for *Rosaceae* (rosaceae.org)) and variant calling was conducted with GATK software (https://gatk.broadinstitute.org). Filtering criteria for the SNPs included a minor allele frequency (MAF) ≥ 0.05, sample sequence completeness at SNP sites of ≥70%, and that markers must be bi-allelic. To minimize redundancy in the resultant SNP dataset, LD pruning was performed using PLINK software (v. 1.07; https://zzz.bwh.harvard.edu/plink) with parameters set to a window size of 50, a step size of 5 and an *r*^2^ threshold of 0.5. Population structure analysis of the filtered SNP data was carried out using Admixture software (v1.3.0, https://github.com/BGI-shenzhen/PopLDdecay), with cross-validation for cluster size (*K*; 1–20) and the selection of *K* = 13 for the lowest CV. To assess the genetic linkage among SNP markers in the population, LD decay was analyzed with the parameters MAF ≥ 0.05, MaxDist = 500, Miss = 0.3, and OutPairLD = 5. GWAS was conducted to analyze potential relationships between the retained SNPs and CR traits. The kinship matrix (K matrix) was calculated using TASSEL software (v5.2.59, https://tassel.bitbucket.io) using both GLM and MLM. To limit the number of false positives arising from multiple testing, candidate SNPs showing *P* < 1/*N* (where *N* is the total number of independent SNPs tested), i.e. *P* < 7.59 × 10^−6^, were selected (hereafter referred to as the ‘suggestive’ threshold). A Bonferroni corrected (α = 0.05) threshold, corresponding to *P* < 0.05/*N*, or *P* < 3.80 × 10^−7^, was also established to highlight the most robust associations.

### Assessment of KASP molecular markers for SNP-associated CR phenotypes

KASP markers for significant CR-associated SNP loci (±150 bp) identified from the GWAS analysis described above were produced by LGC Biosearch Technologies (Shanghai, China). Primers were designed using Primer Premier 5.0 (Premier Biosoft, San Francisco, CA, USA). The fluorescent labels used for the allele-specific forward primers were fluorescein amidite (FAM) and 2′-chloro-7′phenyl-1,4-dichloro-6-carboxy-fluorescein (VIC). Further details of the KASP reaction can be found in Table S2. DNA was extracted from young leaf tissues following the CTAB method. The extracted DNA was diluted in nuclease-free water to a working concentration of 1 to 9 ng/μl, as recommended for KASP assays. Genotyping with the KASP markers was performed using the CFX Connect™ real-time fluorescence quantitative PCR system (Bio-Rad, USA). The PCR reaction volume was 5 μl, consisting of 2.5 μl of 2× KASP master mix, 1.25 μl of primer mix, and 1.25 μl DNA template. Positive controls consisted of samples from accessions in which the genotypes of selected SNP loci had been confirmed by Sanger sequencing. Negative controls omitted the template DNA. The PCR program was as follows: an initial denaturation at 95°C for 10 min followed by denaturation at 95°C for 20 s and annealing/extension at 61°C to 55°C for 60 s (10 cycles); denaturation at 95°C for 20 s and annealing/extension at 55°C for 60 s (27 cycles). The final fluorescence signal was acquired at 25°C for 30 s. The FAM and VIC fluorescence intensities were recorded using CFX Manager™ software (Bio-Rad, USA). Samples with predominant FAM or VIC signals were identified as homozygous for the tested allele, those with equal signal proportions were classified as heterozygous, while samples that could not be reliably classified were marked as undetermined [22]. The accuracy of the KASP genotyping assays was validated data using Sanger sequencing of 20 randomly selected accessions (all confirmed).

To assess the utility of the KASP markers for the prediction of CR phenotypes, a Pearson correlation analysis of their associations with CR phenotypes was performed with significance testing using TASSEL software v5.2.90 [29]. For the diverse panel of peach accessions, CR was stratified into two primary categories: low-CR (<400 h) and mid-to-high CR (≥400 h), with particular attention given to the extreme subsets within each (CR ≤ 300 h and CR ≥ 900 h, respectively).

### Identification and expression analysis of candidate CR-associated genes

Based on LD decay characteristics, genomic regions spanning 150 kb upstream and downstream of SNPs associated with CR phenotypes were selected to identify potential candidate genes. The relative expression of the candidate genes between low-CR and high-CR cultivars was examined by quantitative real-time PCR (qRT-PCR) to prioritize candidate genes potentially involved in CR regulation. The qRT-PCR was carried out using the Hieff® qPCR SYBR Green Master Mix (Yeasen, Shanghai, China), in accordance with the manufacturer's instructions. Gene-specific primers were designed using Primer Premier 5.0 software, with the standard qRT-PCR design principles. The *PpTEF* gene was used as an internal reference (primer sequences listed in Table S3). Each assay included three biological replicates and three technical replicates. Relative gene expression levels were calculated using the 2^(−ΔΔCt) method [30].

Given the limited sample size (only three varieties per high- and low-CR group), the statistical power for detecting differences was constrained. Therefore, to conservatively identify core candidate genes associated with CR variation while prioritizing robust biological effects over marginal statistical significance, we employed an effect-size-informed strategy within a unified linear mixed model framework. For each gene, expression was modeled as a function of CR group (high/low) and dormancy stage, including their interaction term (group × stage). To focus on biologically substantial patterns, we applied the following consecutive and stringent criteria based on Cohen's [31, 32] widely accepted benchmarks: (i) significant interaction effect: the ‘group × stage’ interaction had to be statistically significant (*P* < 0.05) with a large effect size (partial η^2^ > 0.14), indicating that the expression pattern across stages differed fundamentally between CR groups; and (ii) large effect at dormancy stage transitions: for genes meeting the first criterion, we identified the specific consecutive dormancy stage transition where the difference in expression change between the high- and low-CR groups was largest (|Cohen's *d*| > 0.8, representing a large effect size). This phase was defined as the key biological period of divergent expression response. (iii) High within-group consistency: genes were required to show highly consistent expression trends within each CR group across stages, with absolute Pearson's correlation coefficients (|*r*|) > 0.75 for both the high- and low-CR groups. Only genes satisfying all three criteria were designated as core candidate genes. Differential expression between CR groups was subsequently statistically tested and visualized specifically for the key phase identified in Step 2.

Homologies of candidate gene coding sequences were searched for using BLASTp (https://blast.ncbi.nlm.nih.gov/Blast.cgi) with default settings. The candidate genes and close homologues were functionally annotated using the Gene Ontology (GO; http://geneontology.org) and the Kyoto Encyclopedia of Genes and Genomes (KEGG; https://www.kegg.jp) databases.

## Supplementary Material

Web_Material_uhag069

## Data Availability

All data are openly accessible. The main data supporting the findings of this study are available within the paper and its Supplementary materials. The raw whole-genome resequencing data for the 213 peach accessions will be deposited in the publicly accessible databases upon manuscript acceptance. The associated accession number(s) will be provided during the proofing process.
