# Supplementary material for: Uncovering novel loci and developing functional Kompetitive Allele Specific PCR markers for chilling requirement in peach via genome-wide association study
Source: Hortic Res. 2026 Mar 5;13(6):uhag069. doi: 10.1093/hr/uhag069 (PMC13253349; doi:10.1093/hr/uhag069)
Supplement: Web_Material_uhag069 [file web_material_uhag069.zip › Supplementary figures-a clean version(1).docx]

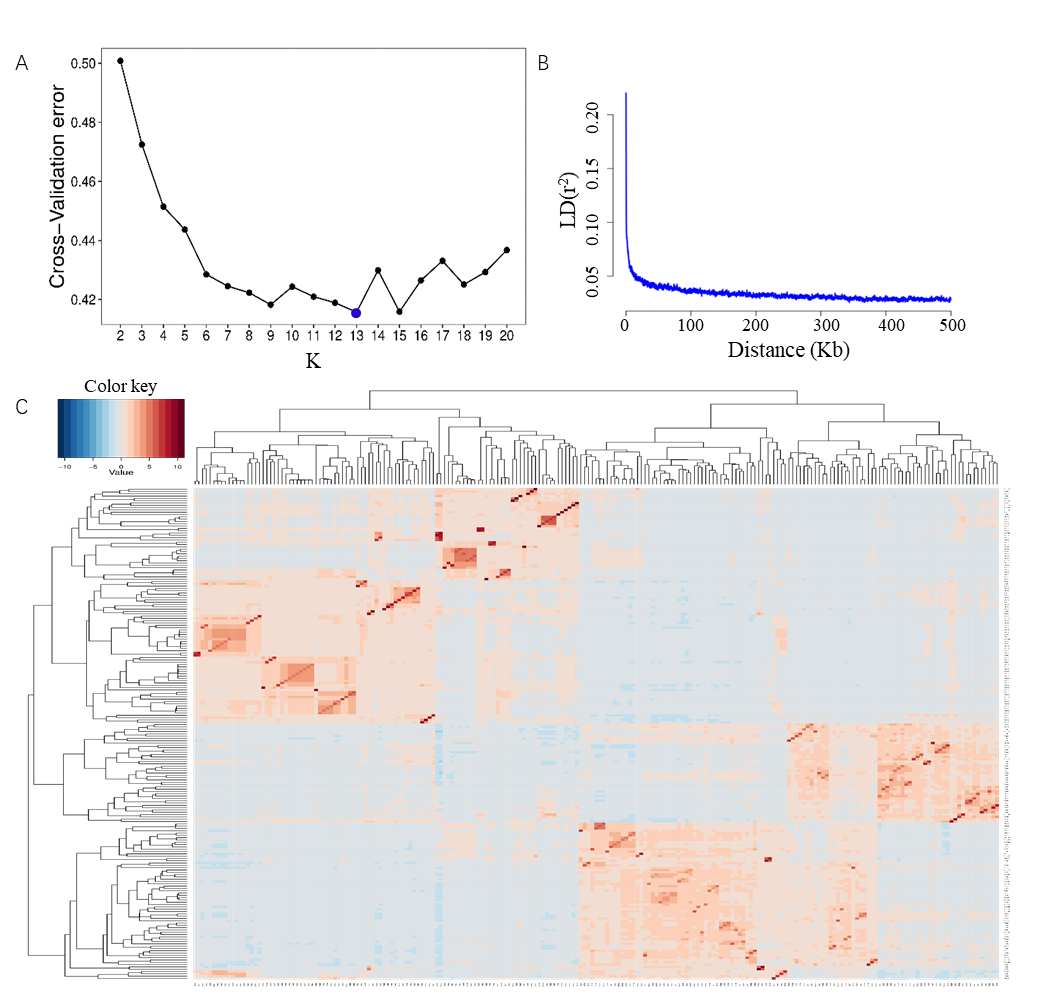


Figure S1. Analysis of Cross-validation error (CV Error), Linkage Disequilibrium (LD) Decay, and Kinship Relationships in the GWAS Population. (A) Cross-validation error (CV Error) plot. The horizontal axis represents different K values, while the vertical axis shows their associated CV error. This analysis aids in determining the optimal K value for population structure analysis. The blue dot indicates the minimum error at K=13. (B) Linkage disequilibrium (LD) decay plot. The horizontal axis represents the physical distance between SNPs, while the vertical axis represents the LD coefficient (a measure of the correlation between SNPs). This plot demonstrates the rate of LD decay as a function of distance. (C) Kinship matrix heatmap. The horizontal and vertical axes represent the sample IDs, while the color gradient reflects the degree of genetic relatedness between individuals. The legend for genetic relatedness (Color key=-10~10) is presented at the top left of the panel where red colored sample intersects indicate a closer genetic relationship.


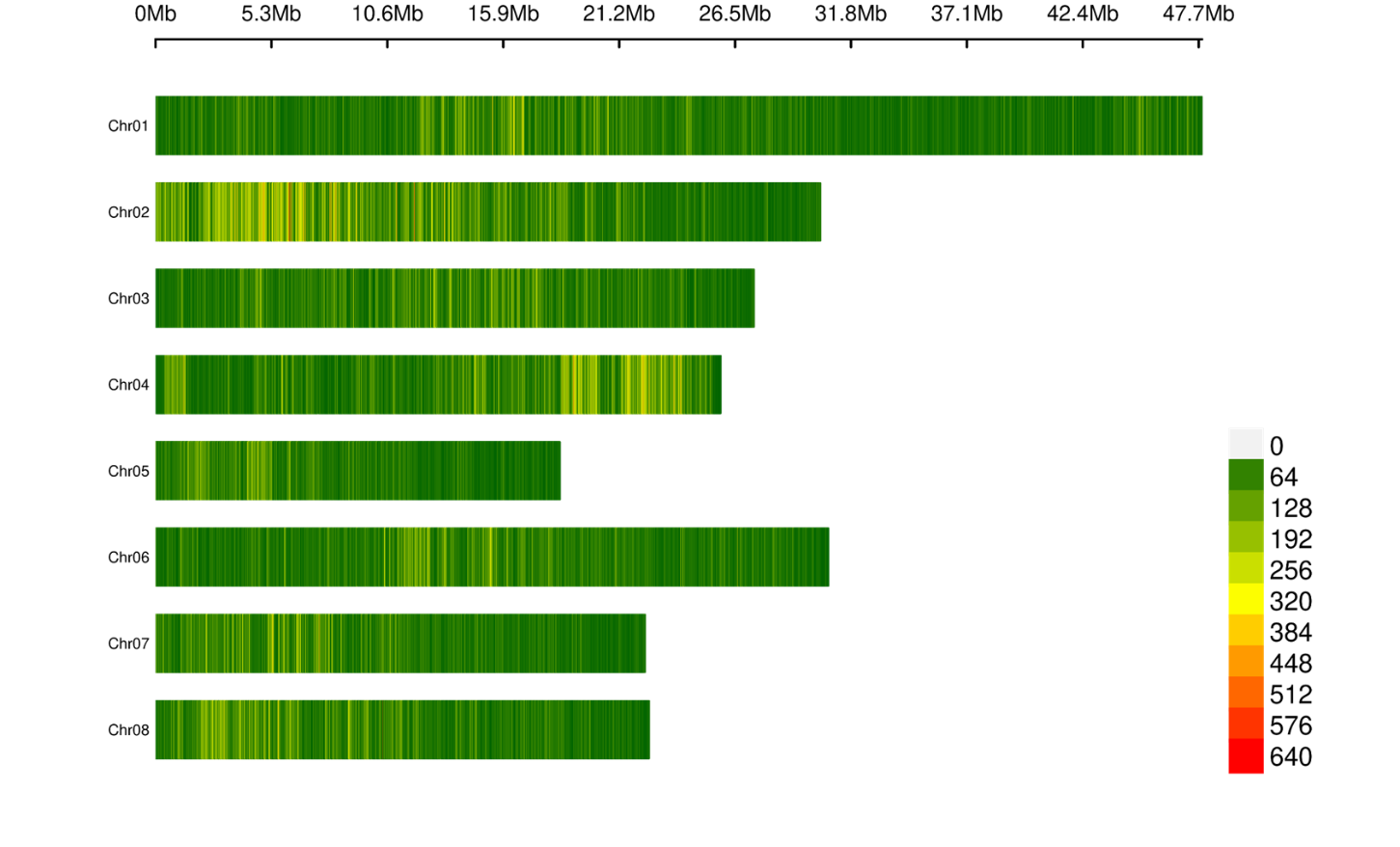


Figure S2. SNP density per chromosome. Each panel displays subplots for chromosomes 1-8 of Peach representing the number of SNPs within a 10kb window size. The horizontal axis shows the chromosome length (Mb), while the different colors depict SNP density (color-mapped as indicated in the color legend to the right.


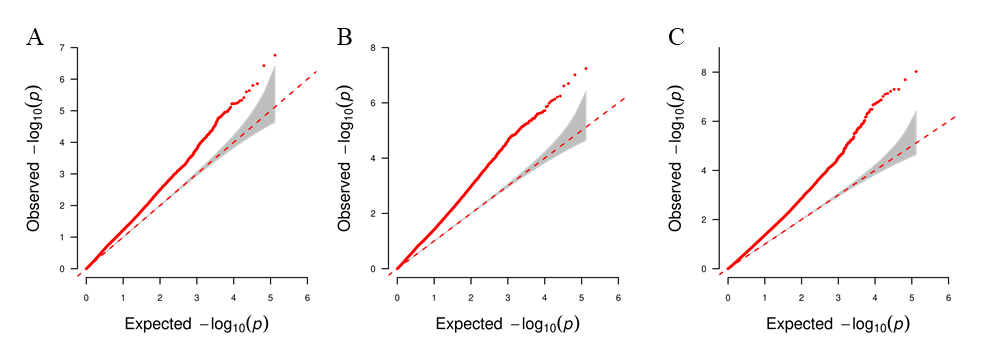


Figure S3. Quantile-quantile (QQ) plots of the GWAS p-values for CR. Panels A, B, and C correspond to the analyses for the 2019–2020, 2021–2022, and 2023–2024 seasons, respectively, based on best linear unbiased prediction values. The underlying linear mixed model included a kinship matrix to control for genetic relatedness. The red dots represent the distribution of observed p-values against the expected null distribution (diagonal line). The gray-shaded band represents the 95% confidence interval. The consistent upward shift across seasons indicates a mild enrichment of low p-values, which is expected for a complex polygenic trait and suggests effective control of false positives.


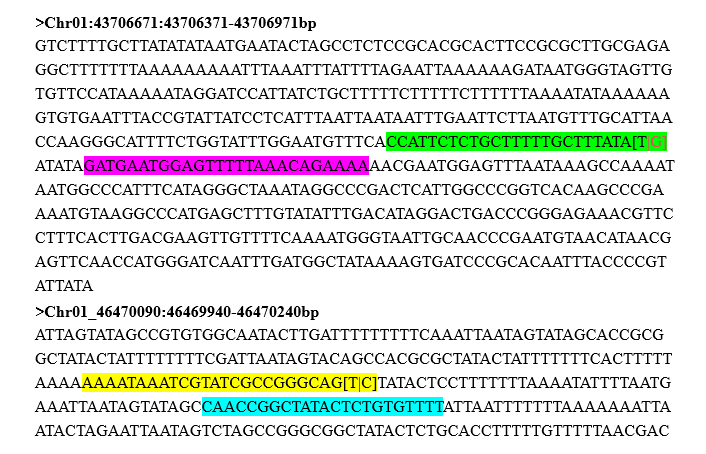


Figure S4. Detailed Primer Sequences for the Two KASP Markers, Chr01:43,706,671 and Chr01:46,470,090. The KASP markers for SNPs at Chr01:43,706,671 and Chr01:46,470,090 are based on the surrounding upstream and downstream sequences Chr01: 43706371-43,706,971 bp and Chr01: 46,469,940-46,470,240 bp, respectively. Both KASP primers were designed in the forward direction according to the 5'-3' sequence. The green-highlighted sequence represents the forward primer for Chr01:43,706,671. The purple-highlighted sequence indicates the shared reverse primer for Chr01:43,706,671. The yellow-highlighted sequence represents the forward primer for Chr01:46,470,090. The blue-highlighted sequence indicates the shared reverse primer for Chr01:46,470,090.


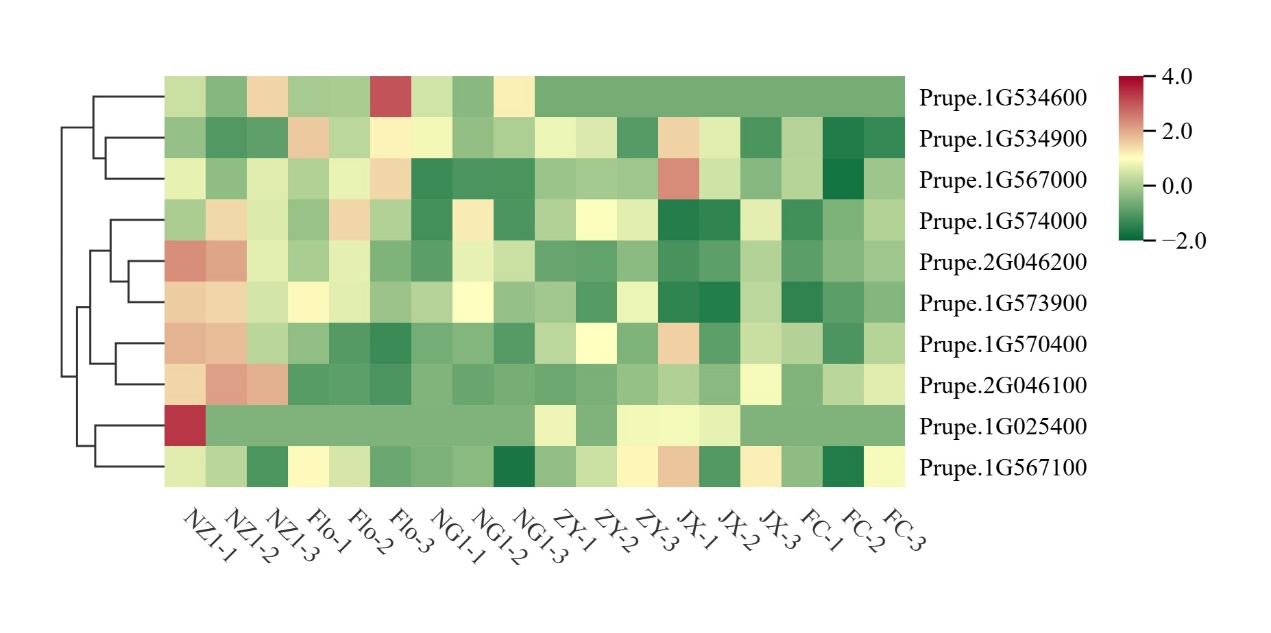


Figure S5: Heatmap of the relative expression levels of 10 CR-Associated genes in peach varieties of different CRs. NZ1, Flo, and NGT1 are three low-CR varieties with CRs of 189 h, 232 h, and 283 h, respectively, and ZY14-2, JX, FC are three high-CR varieties with CRs of 669 h, 686 h, and 1037 h, respectively. The suffixes -1, -2, -3 represent different endodormancy stages of onset, mid-endodormancy and release, respectively. The expression levels of genes are shown relative to their expression in the Low-CR cultivar, NZ1-1, and color mapped according to the legend depicted to the right of each panel.


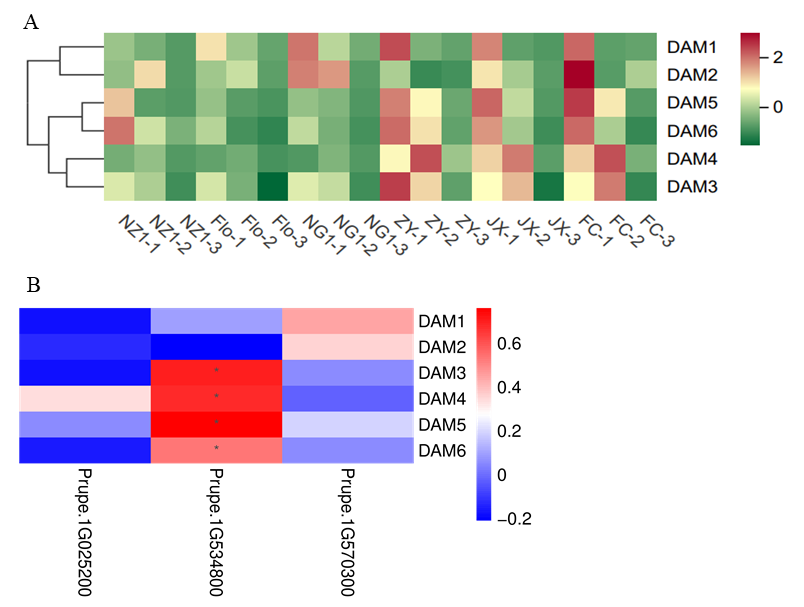


Figure S6. Expression and co-expression analysis of DAM and candidate CR-associated genes during endodormancy. (A) Relative expression levels of *PpDAM1–6* in low and high CR peach varieties. The cultivars used and bud stages utilized are as described in Figure S5. The expression levels of genes are shown relative to their expression in the Low-CR cultivar, NZ1-1, and are color-scaled as shown in the legend. (B) Co-expression analysis between *PpDAM1–6* and three candidate CR-associated genes (*Prupe.1G534800*, *Prupe.1G570300*, *Prupe.1G025200*). Red and blue mapped intersects indicate positive and negative correlations, respectively. Asterisks (*) denote statistically significant correlations (p < 0.05).
