# Supplementary figures and images for: Uncovering novel loci and developing functional Kompetitive Allele Specific PCR markers for chilling requirement in peach via genome-wide association study
Source: Hortic Res. 2026 Mar 5;13(6):uhag069. doi: 10.1093/hr/uhag069 (PMC13253349; doi:10.1093/hr/uhag069)

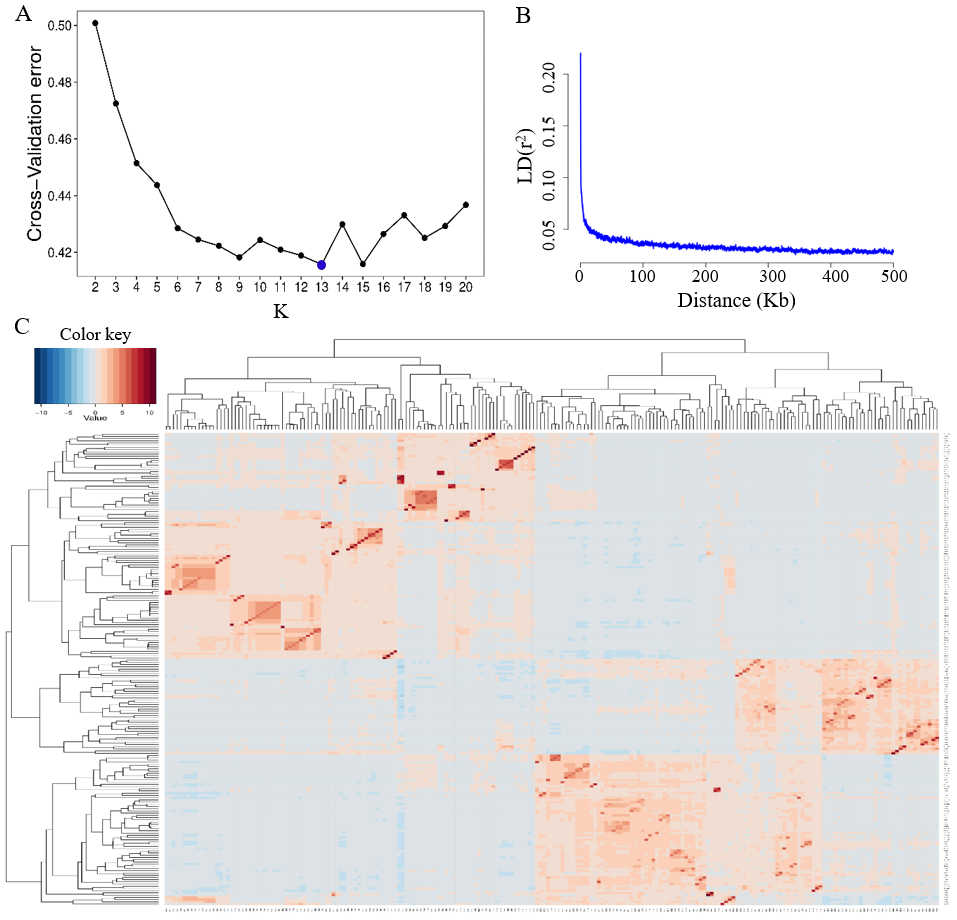

Supplement: Web_Material_uhag069 [file web_material_uhag069.zip › Figure S1.png]

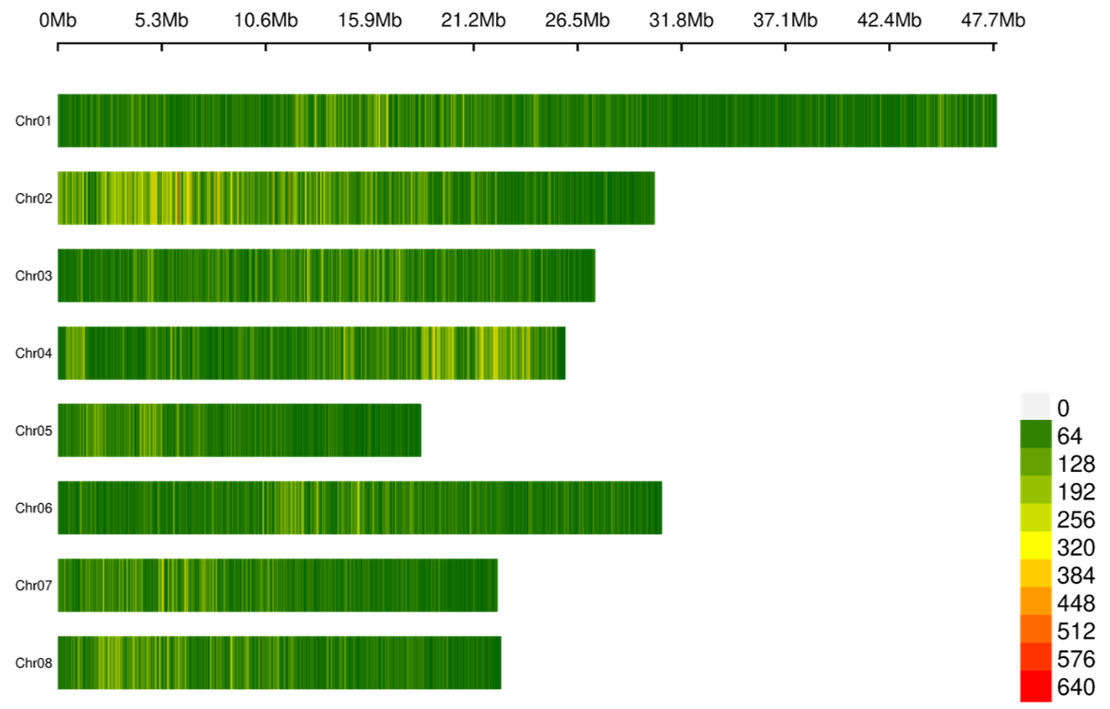

Supplement: Web_Material_uhag069 [file web_material_uhag069.zip › Figure S2.png]

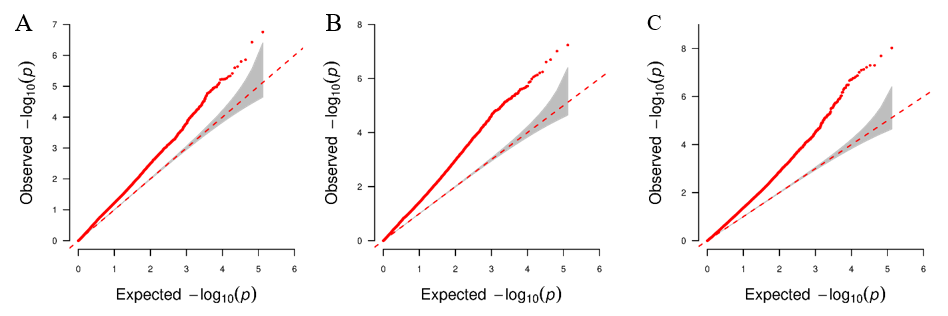

Supplement: Web_Material_uhag069 [file web_material_uhag069.zip › Figure S3.png]

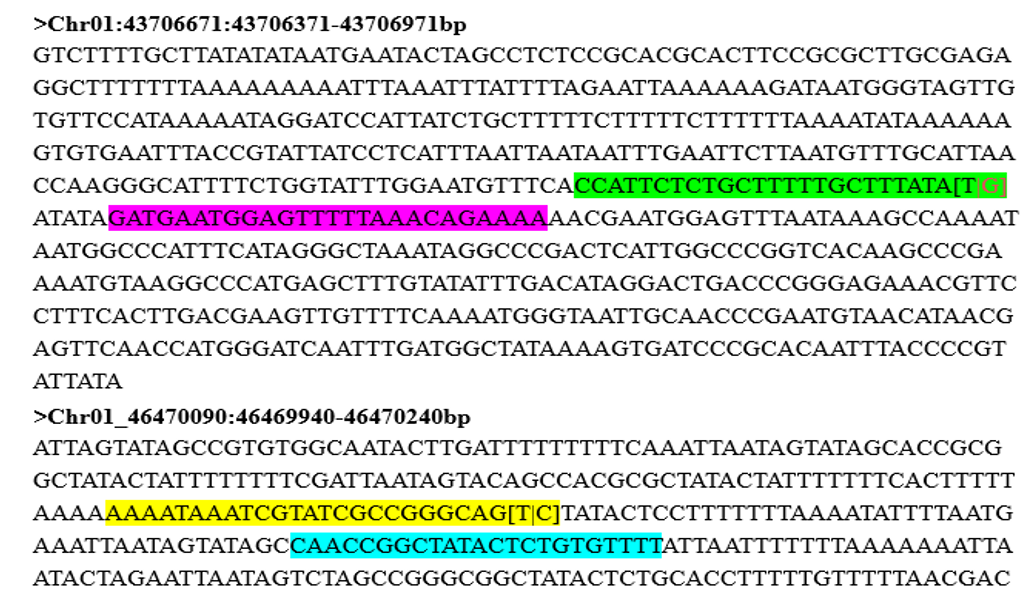

Supplement: Web_Material_uhag069 [file web_material_uhag069.zip › Figure S4.png]

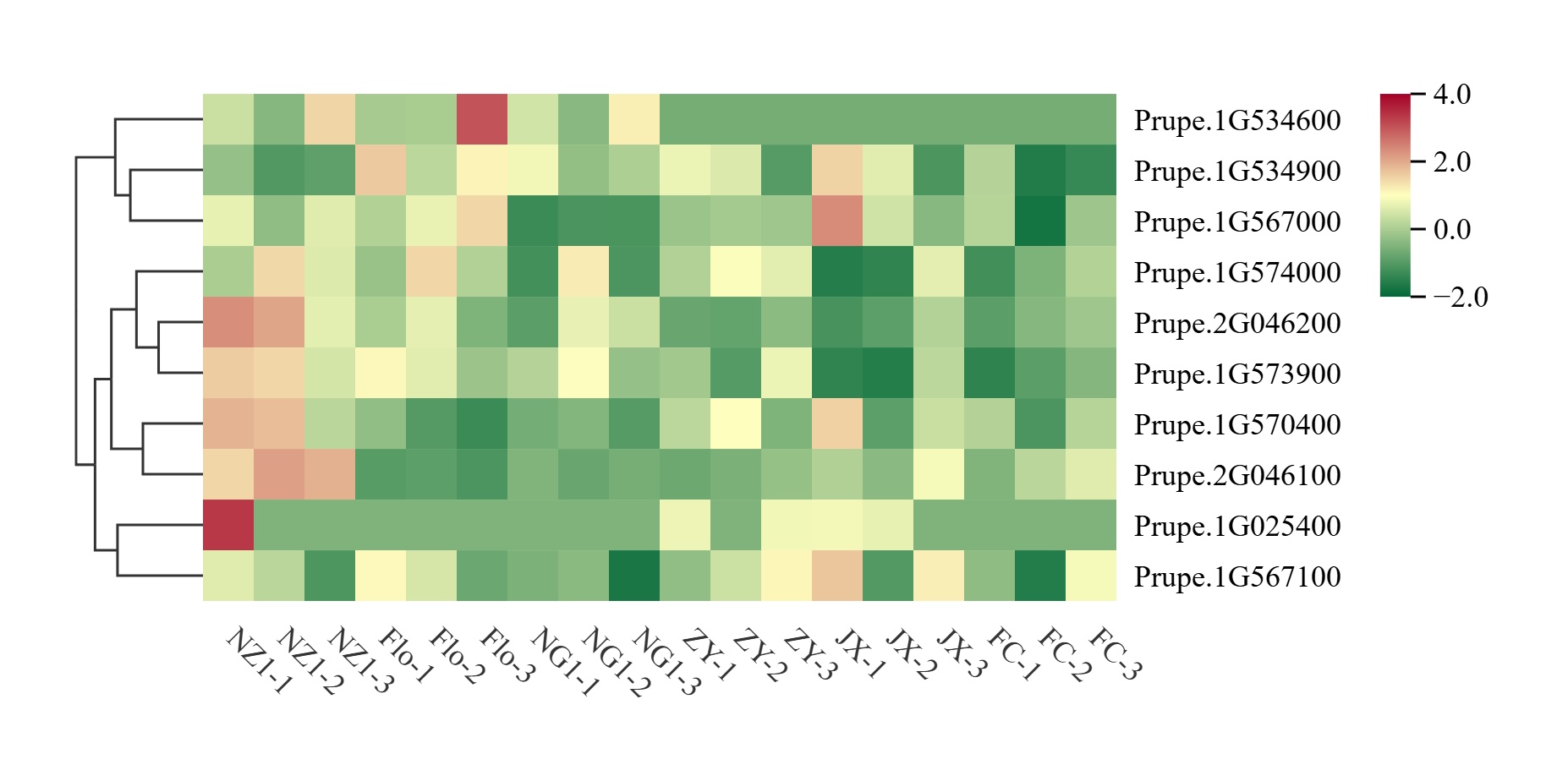

Supplement: Web_Material_uhag069 [file web_material_uhag069.zip › Figure S5.jpeg]

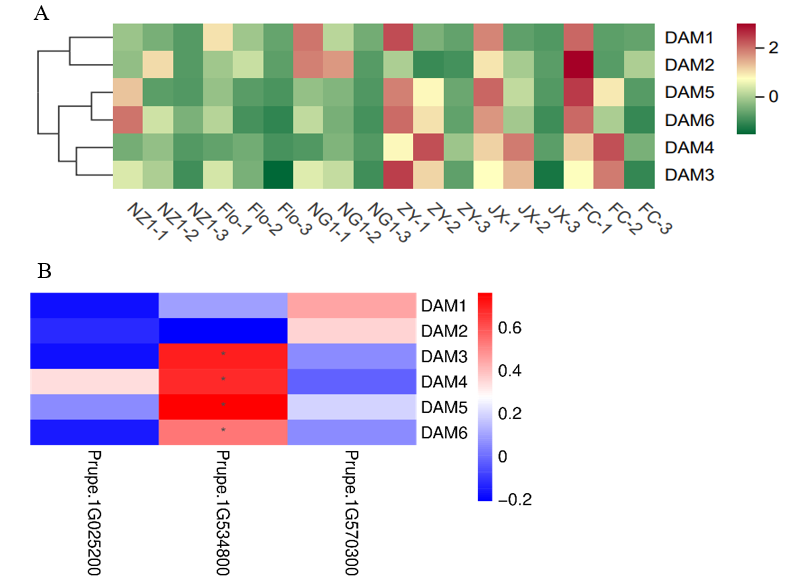

Supplement: Web_Material_uhag069 [file web_material_uhag069.zip › Figure S6.png]
